# A head-to-head comparison of eight unique personality systems in predicting somatization phenomenon

**DOI:** 10.1186/s12888-023-05424-1

**Published:** 2023-12-05

**Authors:** Farzin Rezaei, Khaled Rahmani, Azad Hemmati, Saeid Komasi

**Affiliations:** 1 Department of Psychiatry, Roozbeh Hospital, Tehran University of Medical Sciences, Tehran, Iran; 2 Liver and Digestive Research Center, Research Institute for Health Development, Kurdistan University of Medical Sciences, Sanandaj, Iran; 3 Department of Psychology, University of Kurdistan, Sanandaj, Iran; 4 Neurosciences Research Center, Research Institute for Health Development, Kurdistan University of Medical Sciences, Sanandaj, Iran; 5 Department of Neuroscience and Psychopathology Research, Mind GPS Institute, Kermanshah, Iran

**Keywords:** DSM-5, Health anxiety, ICD-11, Maladaptive personality, Somatic symptom disorder, Temperament

## Abstract

**Background:**

If somatization is an independent personality trait, it is not clear whether it is specific to the temperament or maladaptive spectrum of personality. We aimed at the head-to-head comparison of temperament and maladaptive systems and spectra of personality to predict both somatization and somatic symptom and related disorders (SSRD).

**Methods:**

The samples included 257 cases with SSRD (70.8% female) and 1007 non-SSRD (64.3% female) from Western Iran. The Personality Inventory for DSM-5 (PID-5), Personality Diagnostic Questionnaire-4 (PDQ-4), Temperament and Character Inventory (TCI), Temperament Evaluation of Memphis, Pisa, Paris, and San Diego-Autoquestionnaire (TEMPS-A), Affective and Emotional Composite Temperament Scale (AFECTS), and Positive Affect and Negative Affect Model (PANAS) was used to data collection. A somatization factor plus temperament and maladaptive spectra of personality were extracted using exploratory factor analysis. Several hierarchical linear and logistic regressions were used to test the predictive systems and spectra.

**Results:**

All personality systems jointly predict both somatization and SSRD with a slightly higher contribution for temperament systems. When the temperament and maladaptive spectra were compared, both spectra above each other significantly predicted both somatization (*R*^*2*^ = .407 versus .263) and SSRD (*R*^*2*^ = .280 versus .211). The temperament spectrum explained more variance beyond the maladaptive spectrum when predicting both the somatization factor (change in *R*^*2*^ = .156 versus .012) and SSRD (change in *R*^*2*^ = .079 versus .010).

**Conclusion:**

All temperament and maladaptive frameworks of personality are complementary to predicting both somatization and SSRD. However, the somatization is more related to the temperament than the maladaptive spectrum of personality.

**Supplementary Information:**

The online version contains supplementary material available at 10.1186/s12888-023-05424-1.

## Introduction

Somatic Symptoms and Related Disorders (SSRD) refer to the revised concept of somatoform spectrum disorders proposed in the fifth edition of the Diagnostic and Statistical Manual of Mental Disorders (DSM-5) [1]. SSRD includes several diagnostic classifications such as somatic symptom disorder and illness anxiety disorder. Somatic symptom disorder is characterized by a set of very distressing physical symptoms (usually lasting at least six months) that lead to significant dysfunction as well as severely disproportionate thoughts, feelings, and behaviors about these symptoms [2]. In turn, illness anxiety disorder is a psychiatric condition characterized by excessive concern about having or developing a serious undiagnosed medical illness [2]. Recent cross-cultural studies have reported the prevalence of somatic symptom disorder and illness anxiety disorder at 11–24% [3, 4] and 2–5% [5, 6], respectively. This shows that somatization is a relatively common psychiatric condition in different societies [3–6].

Somatization is a complex psychiatric phenomenon that is referred to as an independent personality trait [7]. Because of the strong and complex association between somatization and personality, a recent review study suggests a pain personality [8]. However, we do not know whether somatization is a phenomenon specific to the temperament or maladaptive spectrum of personality. The results of a fresh review indicated that somatization largely overlaps with both the self-pathology functioning of personality and the negative affectivity domain following Criteria A and B in the Alternative Model for Personality Disorders (AMPD) or the DSM-5 trait model [7]. Criterion A on the DSM-5 trait system refers to the intrapersonal (identity and self-direction) and interpersonal (empathy and intimacy) levels of personality functioning while Criterion B included the maladaptive domains of personality (i.e., negative affectivity, detachment, antagonism, disinhibition, and psychoticism) [9]. These personality domains are the maladaptive poles of the adaptive domains of the Five-Factor Model, including emotional stability, extroversion, agreeableness, conscientiousness, and openness [10]. The structure of the DSM-5 trait model also is relatively similar to the International Classification of Diseases (ICD-11) trait model, including five domains of negative affectivity, detachment, dissociality, disinhibition, and anankastia [11]. The AMPD also proposes six personality disorder (PD) composites including schizotypal, antisocial, borderline, narcissistic, avoidant, and obsessive–compulsive diagnoses that can be alternatives to the traditional ten PD classifications on the DSM-5 Section II including paranoid, schizoid, schizotypal, antisocial, borderline, narcissistic, histrionic, avoidant, dependent, and obsessive–compulsive PDs [2, 9]. Although many studies have tried to examine the associations of the ten DSM-5 PD classifications and domains of the Five-Factor Model with somatization [12–15], there is little evidence for the links between the other three maladaptive personality classification systems (i.e., the DSM-5 and ICD-11 trait systems and the DSM-5 PD composite) and somatization [7].

Similar to the maladaptive spectrum of personality, research on the links between temperament models of personality and somatization has been neglected for many years [16, 17]. Temperament traits, especially in more severe forms, are maladaptive predispositions that make a person vulnerable to both personality disorder and general psychopathology [18]. Some of the most important temperament theories include the affective temperament model [19], the temperament and character model [20, 21], the model of affective and emotional composite temperament (AFECT) [22], and the positive and negative affect/temperament model [23]. The affective temperament model includes five depressive, cyclothymic, hyperthymic, irritable, and anxious temperaments that were originally conceptualized for affective disorders [19]. The temperament and character model includes novelty-seeking, harm avoidance, reward-dependence, and persistence temperaments as well as the character dimensions of self-directedness, cooperativeness, and self-transcendence that were originally conceptualized for somatization [20, 21]. The AFECT model includes six emotional temperaments (volition, anger, inhibition, sensitivity, control, and coping) and twelve affective temperaments [22]. The model proposed by Watson et al. (1988) includes two positive and negative affect/temperament [23]. The complex associations of the temperament models and traits with somatization were addressed by some reports [16, 17, 24–26].

### The present study

It seems that for many years there has been a link between research on the SSRD and studies on personality theories. SSRD is linked to personality psychology not only through common genetic origins with other mental disorders but also through cognitive, emotional, and behavioral aspects [12, 13]. Although maladaptive personality is considered to be the most clinically significant problem for people suffering from SSRD [7, 15, 27], some reports also address the significant role of temperament traits such as harm avoidance, positive temperament, and types of affective temperaments [16, 17, 24–26, 28]. However, less than 15 studies in the last three decades tested the relationship between temperament traits and somatization [16]. There are also some limitations, which were not previously addressed. First, most studies focused on reporting associations between somatization and temperament traits rather than temperament models (i.e., fewer studies reported the predictive power of the models). Second, some temperament traits proposed by different theories strongly overlap (e.g. anxious and depressive temperaments suggested by the affective temperament and AFECT models). Therefore, the possibility of reporting some biased findings is due to the collinearity between predictors and the suppressive effect of regression models. One study [28], however, tried to avoid the effect of these issues on the results by extracting the conjoint structure of several temperament models. Third, few data are comparing the predictive power of temperament and maladaptive systems of personality to predict somatization or SSRD. Fourth, even if research has reported the predictive power of each of the temperament models, attempts to compare the predictive power above and beyond the maladaptive systems of personality such as the criterion-based and trait-based models by the DSM-5 are rare. This means that little knowledge is available to head-to-head compare temperament and maladaptive frameworks of personality to predict somatization or SSRD. If somatization is an independent personality trait [7], it is not clear whether it is specific to the temperament or maladaptive spectrum of personality.

Therefore, we aimed at the head-to-head comparison of eight temperament (i.e., affective temperament model, temperament and character model, affective and emotional composite temperament, and positive and negative temperament) and maladaptive (DSM-5 PD classification, DSM-5 PD composite, DSM-5 trait model, and ICD-11 trait model) systems of personality to predict both somatization and SSRD. We tested the validity and incremental validity of each of the personality models above and beyond each other to predict the criterion variables. We also aimed at the head-to-head comparison of two temperament and maladaptive spectra of personality to predict both somatization and SSRD. Thus, two spectra extracted by exploratory factor analysis above and beyond each other were used to predict the criterion variables.

## Methods

### Design and samples

Participants in this cross-sectional study included 257 cases with SSRD (182 female; 70.8%) and 1007 HCs (648 female; 64.3%) in the west of Iran. The samples were selected from the Kermanshah and Sanandaj cities between April 2020 and August 2021. The population of these cities is about 1.5 million people, most of whom are of Kurdish ethnicity. All samples were adults 18 years old and older, free from psychiatric medications for the last four weeks, and fluent in the Farsi language. Samples of the control group were selected from the general population using public calls in common applications and convenient sampling. The initial general population consisted of 1900 university students, staff in the health sciences centers and other educational institutions, those who were referred to health centers, and housewives. Initially, 82.8% of people completed and returned the questionnaires (*n* = 1581). Questionnaires of 214 people also contained 15 to 90% of the missing data (*n* = 1367). Also, 101 people were excluded from the study due to multiple sclerosis (*n* = 8) or severe epilepsy (*n* = 1), hepatitis (*n* = 4), drug addiction (*n* = 1), cancer (*n* = 1), physical symptoms of coronavirus (*n* = 57), and physical problems from other medical conditions (*n* = 29). Finally, the data of 1266 people were found to be usable. Full details of samples and sampling can be found elsewhere [28].

**Fig. 1:**
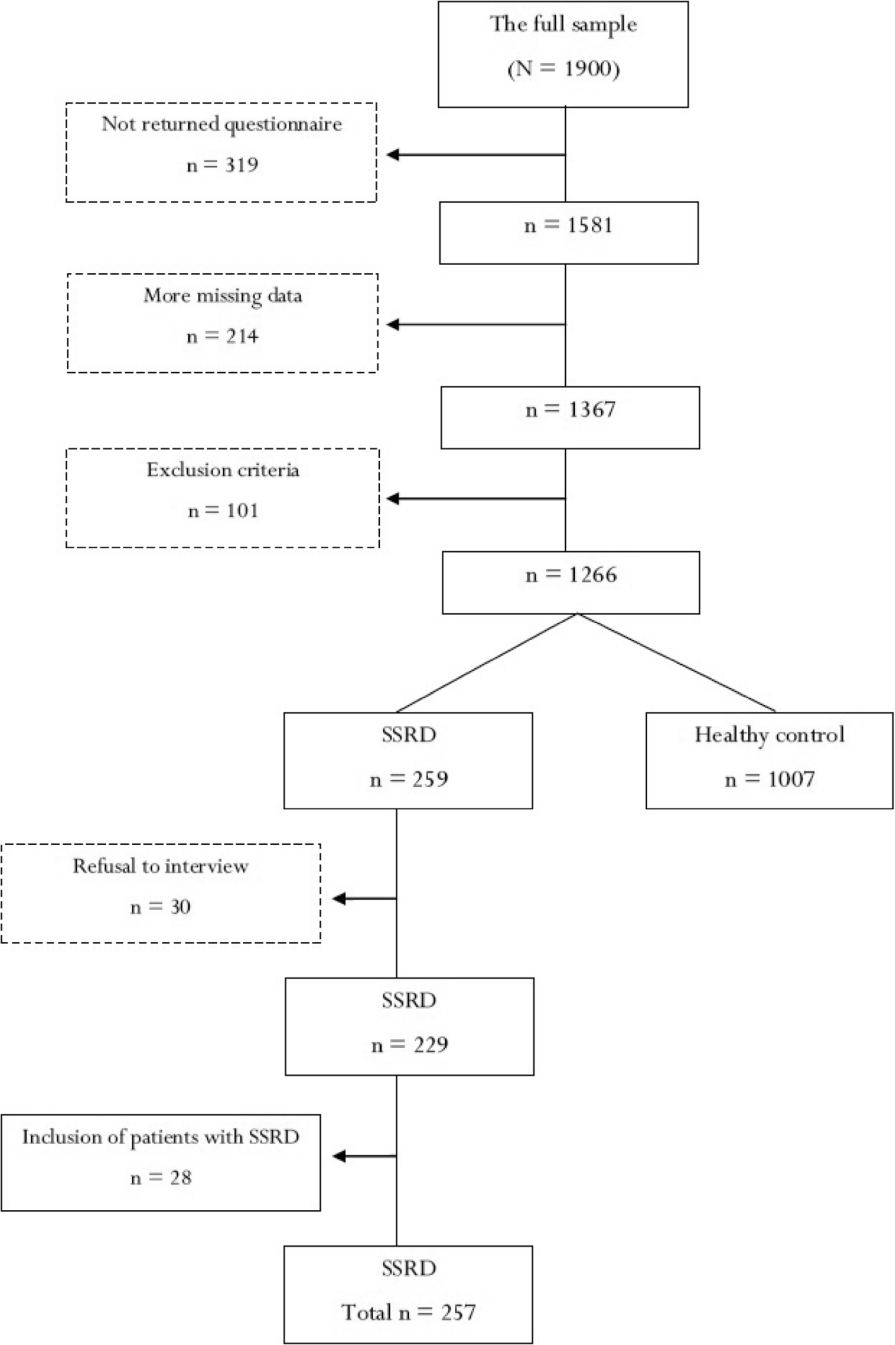
Flowchart of sampling and grouping process

In the next step, cases with SSRD were screened by the subject self-reports and sensitive cutoff scores for the Iranian population including scores higher than 15.5 or 18 on the Screening for Somatic Symptom Disorders (SOMS-7) and the Short Health Anxiety Inventory (SHAI), respectively [29, 30]. Then, the identified cases were assessed using an online diagnostic interview according to DSM-5 Criteria by an expert clinical psychologist. Although 30 people were excluded because they refused to participate in the interview, the diagnosis of SSRD was confirmed for 229 people. Thus, the control group decreased from 1266 to 1007. We then identified 28 patients with SSRD from two psychiatric hospitals in Sanandaj and Kermanshah cities and added them to the sample screened in the previous phase (*n* = 28). Therefore, the cases with SSRD reached 257 people. Figure 1 shows the flowchart of the sampling and grouping process. All data and clinical interviews were conducted by two experienced psychologists. The data were measured using the Personality Inventory for DSM-5 (PID-5; 220 items), the Fourth Edition of the Personality Diagnostic Questionnaire (PDQ-4; 99 items), the Temperament Evaluation of Memphis, Pisa, Paris, and San Diego-Autoquestionnaire (TEMPS-A; 35 items), the Temperament and Character Inventory (TCI; 125 items), the Affective and Emotional Composite Temperament Scale (AFECTS; 60 items), the Positive Affect and Negative Affect Schedule (PANAS; 20 items), SOMS-7 (47 items), the Revised Form of Symptom Checklist-90 (SCL-90-R; 90 items), the Patient Health Questionnaire-15 (PHQ-15; 15 items), and SHAI (18 items). This study is consistent with the Helsinki guidelines and it was approved by the ethics committee of the Kurdistan University of Medical Sciences (IR.MUK.REC.1398.169).

### Data measurement

#### Personality Inventory for DSM-5 *(PID-5)*

This self-report scale was developed by Krueger et al. [31] to assess personality pathology according to DSM-5 Section-III (criterion B of the AMPD). The inventory has 220-item including the five broad domains (negative affectivity, detachment, antagonism, disinhibition, and psychoticism) and 25 lower-order facets (emotional liability, anxiousness, separation insecurity, anhedonia, withdrawal, intimacy avoidance, manipulativeness, grandiosity, deceitfulness, impulsivity, irresponsibility, distractibility, unusual beliefs & experiences, perceptual dysregulation, eccentricity, risk-taking, attention-seeking, callousness, hostility, rigid perfectionism, perseveration, submissiveness, depressivity, restricted affectivity, and suspiciousness). Each item is given a score between zero and three from often false to often true. Except for 16 items (7, 30, 35, 58, 87, 90, 96, 97, 98, 131, 142, 155, 164, 177, 210, 215), other items are scored directly. The higher scores on the scale or subscales present a more severe personality psychopathology [31]. The PID-5 algorithms including 16 to 18 maladaptive facets are also used to calculate the five domains of the ICD-11 trait model [32, 33]. The reliability and validity of this scale in Iranian samples have already been confirmed [32]. In the present study, Cronbach's alpha for the subscales ranged from 0.51 (suspiciousness) to 0.91 (eccentricity and depressivity) and the alpha of the whole scale was 0.98. Details of statistics including Cronbach's alpha, mean, standard deviation, skewness, and kurtosis for all subscales can be seen in Table S1.

#### Personality Diagnostic Questionnaire-4 (PDQ-4)

This 100-item self-report questionnaire was designed by Bagby and Farvolden [34] to diagnose symptoms of personality disorders. This dimensional tool evaluates the symptoms of 12 classifications including paranoid, schizoid, schizotypal, antisocial, borderline, narcissistic, histrionic, avoidant, dependent, obsessive–compulsive, depressive, and negativistic personality disorders. All questions are scored directly (No = 0 and Yes = 1) and the total score is between zero and 100. The higher scores on the questionnaire or subscales show more severe personality pathology [34]. The Persian version of the scale has 99 items with acceptable reliability and validity in Iranian samples [35]. According to the diagnostic classification presented in the DSM-5, two subscales of depressive and negativistic PDs were excluded from the present study. In the present study, Cronbach's alpha for the subscales ranged from 0.49 (schizoid) to 0.70 (dependent), and the alpha of the whole scale was 0.92. Details of statistics for all subscales can be seen in Table S2.

#### Temperament Evaluation of Memphis, Pisa, Paris, and San Diego-Autoquestionnaire (TEMPS-A)

The long form of this 110-item questionnaire was designed by Akiskal et al. [36] to measure five affective temperaments. The original and Persian short forms, respectively, have 39 and 35 items (Yes = 1 and No = 0) to assess depressive, cyclothymic, hyperthymic, irritable, and anxious temperaments. These subscales are measured using 8, 7, 8, 6, and 6 items, respectively. All items are scored directly, and the higher scores on the temperaments (except hyperthymic) present more severe predispositions toward psychopathology. Khalili et al. [37] confirmed the reliability and validity of the Persian version of TEMPS-A in the Iranian sample. In the present study, Cronbach's alpha for the subscales ranged from 0.51 (cyclothymic) to 0.80 (depressive), and the alpha of the whole scale was 0.83. Details of statistics for all subscales can be seen in Table S3.

#### Temperament and Character Inventory (TCI)

Cloninger designed the TCI to measure four temperaments (novelty-seeking, harm avoidance, reward-dependence, and persistence) and three character (self-directedness, cooperativeness, and self-transcendence) traits [38]. The TCI is a dimensional self-report tool with four versions of 56, 125, 140, and 240 items. The longer versions of TCI are not only strongly affected by cultural differences, but there has been no official attempt to validate these versions in Iran [28]. Therefore, the 125-item version was used in the current study. The subscales are evaluated using 20, 20, 15, 5, 25, 25, and 15 items, respectively. The answers are "yes/no" and 64 items are scored in reverse. Although very low or high scores on the temperament traits present more severe predispositions toward psychopathology, the higher scores on the character traits of cooperativeness and self-directedness show more adaptive behaviors. The TCI has acceptable reliability and validity among Iranian samples [39]. In the present study, Cronbach's alpha for the subscales ranged from 0.43 (reward dependence) to 0.78 (harm avoidance), and the alpha of the whole scale was 0.66. Details of statistics for all subscales can be seen in Table S4.

#### Affective and Emotional Composite Temperament Scale (AFECTS)

This questionnaire, prepared by Lara et al. [22] has two parts to evaluate the emotional and affective temperaments. The emotional section is a 7-point bipolar scale that covers six subscales. So, the score on each subscale is between 8 and 56. The components of this section include volition (items 1–8), anger (items 9–16), inhibition (items 17–24), sensitivity (items 25–32), coping (items 33–40), and control (items 41–48). Although higher scores on anger, inhibition, and sensitivity show maladaptive predispositions, the higher scores on volition, coping, and control show more adaptive temperaments. The second part contains 12 descriptive items to assess 12 affective temperaments (depressive, anxious, apathetic, cyclothymic, dysphoric, volatile, obsessive, euthymic, hyperthymic, irritable, disinhibited, and euphoric). Each item is scored on a 5-point Likert scale (1 = it does not look like me at all to 5 = it looks exactly like me). The higher scores on the affective part present more severe predispositions toward psychopathology [22]. The acceptable validity and reliability of this questionnaire in Iranian samples have been previously reported [40]. In the present study, Cronbach's alpha for the subscales ranged from 0.59 (control and sensitivity) to 0.91 (volition). Also, the internal consistency of the emotional (α = 0.89) and affective parts (α = 0.73) was acceptable. Details of statistics for all subscales can be seen in Table S5.

#### Positive Affect and Negative Affect Schedule (PANAS)

This 20-item scale is designed to assess both personality traits (in your lifetime…) and mental states (currently…). Half of the questions (10 items) evaluate positive temperament or affect and the other half evaluate negative temperament or affect. All answers are graded on a five-point Likert scale directly from not at all (1 point) to very much (5 points). The construct and concurrent validity of this tool are acceptable and its reliability using Cronbach's alpha for both positive and negative affect subscales was reported as 0.88 and 0.87 respectively [23]. The reliability and validity of this checklist have already been reported as acceptable in Iranian samples [41]. In the present study, the Cronbach's alpha for both the positive and negative subscales was 0.85. Details of statistics for all subscales can be seen in Table S6.

#### Screening for Somatic Symptom Disorders (SOMS-7)

This 47-item questionnaire was developed by Reif et al. [42] to evaluate the severity of the somatic signs/symptoms during the last seven days. Each item is scored directly on a 4-point Likert scale (never = 0 and always = 3; total between zero and 141). The subscales include pain, cardiovascular and respiratory symptoms, gastrointestinal and urologic symptoms, neurological functioning symptoms, and musculoskeletal symptoms. These components are measured using 17, 17, 10, and 3 items, respectively. The higher scores on the questionnaire or subscales present more severe somatic symptoms. The validity and reliability of this questionnaire have been reported as acceptable in normal and clinical populations of Iran [29]. The internal consistency of the whole scale was acceptable in the present study (α = 0.94). Details of statistics for the SOMS-7 and other somatization measures can be seen in Table S7.

#### Revised Form of Symptom Checklist-90 (SCL-90-R)

The SCL-90-R questionnaire which has 90 items to assess psychopathological symptomatology was designed and revised by Derogatis et al. [43, 44] to assess the symptoms of mental disorders. The SCL-90-R subscales include somatization, obsessive–compulsive disorder, depression, anxiety, hostility, phobic anxiety, interpersonal sensitivity, paranoid ideation, psychoticism, and six additional items. The score of each item is determined on a five-point Likert scale from no discomfort to very severe discomfort (score 0 to 4). The higher scores on the total items or subscales show more severe psychopathological symptomatology [43, 44]. The reliability and validity of this checklist have already been reported as acceptable in Iranian samples [45, 46]. In the present study, we used only the somatization subscale with Cronbach's alpha equal to 0.90.

#### The Patient Health Questionnaire-15 (PHQ-15)

The PHQ-15 is a self-report tool for measuring the severity of somatic symptoms during the last week. This questionnaire has no subscales and higher scores indicate more severe physical symptoms. Each of the items on the questionnaire is scored on a three-point scale from "not at all" (zero) to "a lot" (two) and the total score ranged between zero and thirty. The reliability and validity of PHQ-15 have already been confirmed in Persian samples [47]. In the present study, Cronbach's alpha for the scale was acceptable (α = 0.84).

#### Short Health Anxiety Inventory (SHAI)

This questionnaire was designed by Salkovskis et al. [30] to assess health anxiety. Each item is scored directly on a 4-point Likert scale (very low = 0 and very high = 3). Therefore, the total score on the scale is between zero and 54. The SHAI has 18 items to evaluate the three subscales including rumination, probability of disease, and negative outcome. These subscales are measured using 7, 7, and 4 items, respectively. The higher scores on the questionnaire or subscales present more severe health anxiety. The scale has acceptable reliability and validity among Iranian samples [48]. In the present study, Cronbach's alpha for the scale was acceptable (α = 0.84).

### Analytic plan

Before performing parametric statistical methods, the data was screened for the non-violation of statistical assumptions such as normality (skewness and kurtosis between − 1 and + 1). There were two criterion variables including categorical and dimensional somatization. Classification of somatization was revealed in two groups without and with SSRD. To extract the conjoint structure of dimensionally measured somatization, we planned an exploratory factor analysis (EFA) with maximum likelihood estimations on all four scales related to SSRD including SOMS-7, SCL-90 somatization, PHQ-15, and SHAI. We identified a unidimensional factor of somatization with an eigenvalue higher than 1, the details of which are reported in the results section.

In the next step, we tested Pearson's correlation coefficients between the unidimensional factor of somatization and all temperament and maladaptive domains and traits. Then, we used hierarchical linear multiple regression techniques in which all temperament and maladaptive systems of personality were entered as blocks to predict the somatization factor. We entered each of the personality systems once in the first block and again in the second block (i.e., above and beyond the other systems) to calculate both *R*^*2*^ and changes in *R*^*2*^ (incremental validity) in all linear regressions. By calculating the changes in *R*^*2*^, we intended to determine how much additional variance each personality system is explaining in the outcome.

When the two groups non-SSRD and SSRD were compared, the independent t-test results showed that the groups were significantly different in most of the temperament and maladaptive traits. We used hierarchical logistic regression techniques in which all temperament and maladaptive systems of personality were entered as blocks to predict the SSRD. We again entered each of the personality systems once in the first block and again in the second block to calculate both pseudo-*R*^*2*^ (the Nagelkerke static) and changes in *R*^*2*^ in all logistic regressions.

In the final step, we aimed to identify the conjoint structure of both the temperament spectrum and the maladaptive spectrum of personality to predict dimensional and categorical somatization. We planned two EFA with maximum likelihood estimations on both temperament scales (TEMPS, TCI, AFECTS, and PANAS) and maladaptive personality scales (PID-5 and PDQ-4). To identify the simple structure of these homogeneous variables, the factors were rotated using the Promax rotation method. These analyses led to the extraction of a seven-factor temperament spectrum and a five-factor maladaptive spectrum with eigenvalues higher than 1, the details of which can be seen in the results section. These factors were used as temperament and maladaptive spectra to predict both the somatization factor and SSRD. We used hierarchical linear and logistic regression techniques in which two temperament and maladaptive spectra were entered as blocks to predict both the somatization factor and SSRD. We entered each of the five- and seven-factor models once in the first block and again in the second block to calculate both *R*^*2*^ and changes in *R*^*2*^ for all regressions. All statistical analyses were performed using the SPSS software and *p* ≤ 0.05 was considered the significance level.

## Results

The case and control groups contained 830 (66%) and 182 (71%) women, respectively (*p* > 0.05). The mean and standard deviation of the age of all subjects was 33.73 ± 11.29 years. The other demographics and medical, psychiatric, and behavioral history of the groups can be seen in Table S8. To test the conjoint structure of the dimensional scores of somatization measures, EFA extracts a unidimensional factor of somatization with an eigenvalue > 1 (= 2.71). All coefficients were quite strong (ranging from 0.44 to 0.87) and the extracted factor could explain 68% of the variance (χ^2^ = 18.576, *p* < 0.001). Pearson's correlation coefficients showed that the unidimensional factor of somatization is significantly related to most temperament and maladaptive domains and traits (Table S9).

Table 1 shows the head-to-head comparison of all temperament and maladaptive systems or tools as blocks to predict the unidimensional somatization factor. The results of this table show that all temperament and maladaptive personality systems significantly predict the somatization factor in the first block (*R*^*2*^ ranging from 0.149 to 0.376, all *p* < 0.001). In the second block, the incremental validity of all personality systems ranges from 0.003 to 0.236. In more detail, *R*^*2*^ for TEMPS (ranging from 0.103 to 0.236), AFECTS (ranging from 0.033 to 0.166), PANAS (ranging from 0.025 to 0.156), DSM-5 PD composite (ranging from 0.021 to 0.138), ICD-11 trait model (ranging from 0.011 to 0.129), DSM-5 trait model (ranging from 0.011 to 0.118), TCI (ranging from 0.009 to 0.084), and DSM-5 PD classification (ranging from 0.003 to 0.026) beyond the other systems respectively is higher.

**Table 1:** The head-to-head comparison of all temperament and maladaptive personality systems as blocks to predict the somatization factor

Linear regression blocks	Temperament and maladaptive systems or tools (hierarchical multiple linear regressions)															
	DSM-5 classification model		DSM-5 composite model		DSM-5 trait model		ICD-11 trait model		TEMPS		TCI		AFECTS		PANAS	
	*R*^*2*^	*p*	*R*^*2*^	*p*	*R*^*2*^	*p*	*R*^*2*^	*p*	*R*^*2*^	*p*	*R*^*2*^	*p*	*R*^*2*^	*p*	*R*^*2*^	*p*
1: Validity	.149	< .001	.283	< .001	.262	< .001	.273	< .001	.376	< .001	.206	< .001	.307	< .001	.287	< .001
2: Incremental validity																
DSM-5 classification	-	-	.003	.807	.005	.600	.004	.709	.008	.079	.026	< .001	.007	.208	.018	.001
DSM-5 composite	.138	< .001	-	-	.032	< .001	.021	< .001	.023	< .001	.099	< .001	.039	< .001	.060	< .001
DSM-5 trait model	.118	< .001	.011	.002	-	-	.016	< .001	.021	< .001	.082	< .001	.031	< .001	.054	< .001
ICD-11 trait model	.129	< .001	.011	.002	.027	< .001	-	-	.020	< .001	.092	< .001	.037	< .001	.053	< .001
TEMPS	.236	< .001	.116	< .001	.135	< .001	.124	< .001	-	-	.179	< .001	.103	< .001	.115	< .001
TCI	.084	< .001	.022	< .001	.026	< .001	.025	< .001	.009	.010	-	-	.023	< .001	.035	< .001
AFECT	.166	< .001	.062	< .001	.075	< .001	.071	< .001	.033	< .001	.123	< .001	-	-	.073	< .001
PANAS	.156	< .001	.056	< .001	.079	< .001	.067	< .001	.025	< .001	.115	< .001	.053	< .001	-	-

*Abbreviations*: *AFECTS* Affective and Emotional Composite Temperament Scale, *DSM* Diagnostic and Statistical Manual of Mental Disorders, *ICD* International Classification of Diseases, *PANAS* Positive and Negative Affect Schedule, *PID-5* Personality Inventory for DSM-5, *TCI* Temperament and Character Inventory, *TEMPS* Temperament Evaluation of Memphis, Pisa, Paris, and San Diego Autoquestionnaire

A comparison of the mean and standard deviation of all the temperament and maladaptive systems between non-SSRD and SSRD groups is found in Table S10. The results of the independent *t*-test showed that the groups were significantly different in most of the temperament and maladaptive traits. Table 2 shows the head-to-head comparison of all temperament and maladaptive systems or tools as blocks to predict the SSRD. The results of this table show that all temperament and maladaptive systems significantly predict the SSRD in the first block (pseudo-*R*^*2*^ ranging from 0.153 to 0.246, all *p* < 0.001). In the second block, the incremental validity of all personality systems ranges from 0.005 to 0.116. In more detail, pseudo-*R*^*2*^ for AFECTS (ranging from 0.046 to 0.116), TEMPS (ranging from 0.041 to 0.105), PANAS (ranging from 0.020 to 0.085), DSM-5 PD composite (ranging from 0.025 to 0.081), DSM-5 trait model (ranging from 0.015 to 0.081), TCI (ranging from 0.038 to 0.080), ICD-11 trait model (ranging from 0.005 to 0.071), and DSM-5 PD classification (ranging from 0.011 to 0.042) beyond the other systems respectively is higher.

**Table 2:** The head-to-head comparison of all temperament and maladaptive systems of personality as blocks to predict the SSRD

Logistic regression blocks	Temperament and maladaptive systems or tools (hierarchical binary logistic regressions)															
	DSM-5 classification model		DSM-5 composite model		DSM-5 trait model		ICD-11 trait model		TEMPS		TCI		AFECTS		PANAS	
	*R*^*2*^	*p*	*R*^*2*^	*p*	*R*^*2*^	*p*	*R*^*2*^	*p*	*R*^*2*^	*p*	*R*^*2*^	*p*	*R*^*2*^	*p*	*R*^*2*^	*p*
1: Validity	.153	< .001	.223	< .001	.217	< .001	.203	< .001	.241	< .001	.196	< .001	.246	< .001	.196	< .001
2: Incremental validity																
DSM-5 classification	-	-	.011	.355	.017	.094	.021	.035	.017	.088	.037	< .001	.023	.017	.042	< .001
DSM-5 composite	.081	< .001	-	-	.025	.001	.047	< .001	.029	< .001	.078	< .001	.032	< .001	.058	< .001
DSM-5 trait model	.081	< .001	.019	.003	-	-	.015	< .001	.036	< .001	.074	< .001	.037	< .001	.068	< .001
ICD-11 trait model	.071	< .001	.027	< .001	.005	.065	-	-	.025	< .001	.065	< .001	.027	< .001	.053	< .001
TEMPS	.105	< .001	.047	< .001	.060	< .001	.063	< .001	-	-	.084	< .001	.041	< .001	.066	< .001
TCI	.080	< .001	.051	< .001	.053	< .001	.058	< .001	.038	< .001	-	-	.041	< .001	.068	< .001
AFECT	.116	< .001	.055	< .001	.066	< .001	.070	< .001	.046	< .001	.091	< .001	-	-	.070	< .001
PANAS	.085	< .001	.031	< .001	.047	< .001	.046	< .001	.021	< .001	.068	< .001	.020	< .001	-	-

The pseudo *R* squares (*R*^2^) are according to the Nagelkerke static

*Abbreviations*: *AFECTS* Affective and Emotional Composite Temperament Scale, *DSM* Diagnostic and Statistical Manual of Mental Disorders, *ICD* International Classification of Diseases, *PANAS* Positive and Negative Affect Schedule, *PID-5* Personality Inventory for DSM-5, *TCI* Temperament and Character Inventory, *TEMPS* Temperament Evaluation of Memphis, Pisa, Paris, and San Diego Autoquestionnaire, SSRD: somatic symptom and related disorders

Using the conjoint EFA with maximum likelihood estimations and Promax rotations, we extracted seven temperament factors with eigenvalues > 1 (ranging from 1.002 to 8.967). All coefficients were quite strong (ranging from 0.36 to 0.84) and the extracted factors could explain 51% of the variance (χ^2^ = 1296.911, *p* < 0.001). We also extracted five maladaptive factors with eigenvalues > 1 (ranging from 1.693 to 14.876). All coefficients were strong (ranging from 0.27 to 0.83) and the extracted factors could explain 60% of the variance (χ^2^ = 2595.364, *p* < 0.001).

Table 3 shows the head-to-head comparison of two temperament and maladaptive spectra (seven- and five-factor spectra) as blocks to predict both somatization factor and SSRD. When the seven-factor temperament spectrum was entered in the first block, it had an *R*^*2*^ of 0.407 (*p* < 0.001) for somatization factor and 0.280 (*p* < 0.001) for SSRD, whereas when the five-factor maladaptive spectrum was entered in the first block, it had an *R*^*2*^ of 0.263 (*p* < 0.001) for somatization factor and 0.211 (*p* < 0.001) for SSRD. The seven-factor temperament spectrum explained more variance beyond the five-factor maladaptive spectrum when predicting both the somatization factor (change in *R*^*2*^ = 0.156 versus 0.012) and SSRD (change in *R*^*2*^ = 0.079 versus 0.010).

**Table 3:** The head-to-head comparison of two temperament and maladaptive spectra as blocks to predict both somatization factor and SSRD

The extracted personality models	Somatization factor (hierarchical multiple linear regressions)				SSRD (hierarchical binary logistic regressions)			
	Block 1: Validity		Block 2: Incremental validity		Block 1: Validity		Block 2: Incremental validity	
	*R2*	*p*	*R2*	*p*	*R2*	*p*	*R2*	*p*
Temperament model (7 factors)	.407	< .001	.156	< .001	.280	< .001	.079	< .001
Maladaptive model (5 factors)	.263	< .001	.012	< .001	.211	< .001	.010	.094

The pseudo R squares (R^2^) for the binary logistic regressions are according to the Nagelkerke static

*Abbreviations*: *SSRD* Somatic symptom and related disorders

## Discussion

The present study aimed at the head-to-head comparison of eight temperament and maladaptive systems of personality to predict both somatization and SSRD. We found that all personality systems jointly predict both the somatization factor and SSRD with a slightly higher contribution for temperament models. In more detail, our results showed that temperament systems assessed by TEMPS and AFECTS above and beyond other systems predict both somatization and SSRD well. Previous reports support the associations between these temperament models and somatization [16, 25, 26]. The theory of affective temperaments measured using TEMPS was originally conceptualized for affective disorders by Akiskal et al., [19, 36]. A later report suggested a continuum between affective disorders and somatization [25] that may result from a common genetic origin or comorbidity across these diagnostic spectra [49, 50]. The conceptualization of the temperament model by Lara et al. [22], which is measured using AFECTS, is similar to the model of affective temperaments. Although these systems are predispositions belonging to the normality domain, temperaments are problematic when they appear in a severe form [18]. The problematic temperaments are common in 70% of somatoform patients, which differentiates them from healthy controls [25].

Our results also showed that the DSM-5 PD classification is more weakly related to both somatization and SSRD than other personality systems. The DSM-5 PD classification showed a slight incremental validity beyond the TCI and PANAS, while the incremental validity of all personality systems beyond it was strongly significant. The findings may provide some evidence in support of the replacement of the criterion-based system of PD by the trait-based system proposed in the DSM-5 Section III, at least to predict somatization and SSRD. Section III presents both the PD trait and the PD composite systems that are recently supported by a large body of research [2, 9, 10, 51, 52]. Various reports also support the association between the negative affectivity domain of the DSM-5 trait model and somatization [7, 53, 54]. Similar to the DSM-5 trait model, the ICD-11 trait model includes five maladaptive domains such as negative affectivity [11]. Negative affectivity and somatization link through the higher-order factor of emotional dysfunction according to the Hierarchical Taxonomy of Psychopathology (HiTOP) [54, 55]. However, emotional dysregulation is a transdiagnostic construct that is neglected in the DSM-5 PD classification [2]. However, self-report scales used in the current study cannot fully measure all aspects of the theoretical frameworks and cross-sectional correlations between variables do not provide an understanding of causal relationships. Therefore, more evaluation is needed to ensure the utility of trait-based systems in the diagnosis, intervention planning, and treatment response of patients with SSRD.

The present study also aimed at the head-to-head comparison of the temperament and maladaptive spectra of personality to predict both the somatization factor and SSRD. First, using factor analysis techniques, we identified seven factors on the temperament spectrum and five factors on the maladaptive spectrum of personality. Our results showed that temperament spectrum factors above and beyond maladaptive spectrum factors predict both somatization and SSRD well. The maladaptive spectrum factors also showed significant validity in predicting both somatization and SSRD. However, the maladaptive spectrum factors showed a very slight incremental validity (approximately one percent) beyond the temperament spectrum factors. A recent meta-analysis showed that temperament traits are complex and extensively related to the psychopathology of many mental disorders [17]. Most temperament models were originally developed for the psychopathology of non-PDs rather than PDs. For example, the temperament and character model and the affective temperament model were designed and validated for somatization and affective disorders, respectively [19–21]. Therefore, the strong relationship between temperament and somatization models in the current study is not unexpected. Several reports address well the spectrum associations of temperament and other similar constructs with both personality and general psychopathology [56–59]. Because the maladaptive spectrum factors are also related to both somatization and SSRD regardless of the temperament spectrum factors, the importance of the general factor of psychopathology can be discussed [7, 53, 54].

To our knowledge, the present study is a pioneering attempt to the head-to-head compare several temperament and maladaptive systems of personality to predict both somatization and SSRD. We also used data from the large non-Western sample to compare the temperament and maladaptive spectra of personality to predict both the somatization phenomenon. Despite a large sample size, primary diagnoses by cut scores were validated using a diagnostic interview according to DSM-5 criteria [2]. However, our study is unique due to the use of eight temperament models and maladaptive systems of personality related to somatization. Nevertheless, there were some limitations. The current cross-sectional report includes self-reported personality traits, which cannot address the causal associations between personality and somatization. Although there was no significant difference between the groups in terms of age and sex (see Table S1), group matching was not possible for us due to the large sample size. The number of items in the questionnaires was large, which could have led to a decrease in the accuracy of the sample responses. We used formal PID-5 algorithms to calculate the maladaptive domains of the ICD-11 trait model [33] while more valid scales are available for future studies [60]. Future studies may use shorter research forms such as the 100-item version of PID [61]. The small number of cases with illness anxiety disorder made it impossible for us to study both somatic symptom disorder and illness anxiety disorder. Finally, we did not examine the relationship between the general personality factor [62] and somatization spectrum disorders, which could be the target of future studies.

## Conclusion

The results of the present study showed that all temperament and maladaptive systems of personality are complementary to predicting both somatization and SSRD by a slightly higher contribution for temperament systems. In comparison with the trait-based system of PD proposed in the DSM-5 Section III and the trait model of ICD-11, we found that the criterion-based system of PD is more weakly related to both somatization and SSRD. Although self-report scales cannot fully measure all aspects of a theoretical framework and cross-sectional correlations between variables do not provide an understanding of causal relationships resulting from longitudinal studies, the findings may provide some evidence in support of the replacement of the criterion-based system of PD by the trait-based systems, at least to predict somatization and SSRD. However, future research can provide more data for this claim by testing the utility of trait-based systems in the diagnosis, intervention planning, and treatment response of patients with SSRD.

Interestingly, the results showed that both somatization and SSRD are more related to the temperament spectrum of personality than the maladaptive spectrum. The maladaptive spectrum of personality was expected to be a stronger predictor of somatization and SSRD because this spectrum was measured by self-report scales related to the official classification systems of DSM and ICD. This finding shows that temperament traits contribute significantly to the complexity of the somatization phenomenon, and clinicians should take this into account in the process of diagnosis and treatment planning. Future studies can test the validity of the findings to provide more data on the relationship between personality and somatization psychopathology according to formal classification systems compared to more informal classification frameworks. Future studies also will attempt to determine to what extent the link between the temperament and maladaptive systems of personality and SSRD is influenced by the general factor of personality.

### Supplementary Information

**Additional file 1: Table S1.** The PID-5 trait statistics (*n* = 1264). **Table S2.** The PDQ-4 statistics (*n* = 1264). **Table S3.** The TEMPS-A statistics (*n* = 1264). **Table S4.** The TCI statistics (*n* = 1264). **Table S5.** The AFECTS statistics (*n* = 1264). **Table S6.** The PANAS statistics (*n* = 1264). **Table S7.** The statistics of somatization measures (*n* = 1264). **Table S8.** Demographic information and medical, psychiatric, and behavioral history of the groups. **Table S9.** Correlations between all personality systems and somatization factor. **Table S10.** Mean and standard deviation of personality traits between the groups. 

## Data Availability

The current study data are available on reasonable request to S.K., S_komasi63@yahoo.com.
