# Supplementary material for: A head-to-head comparison of eight unique personality systems in predicting somatization phenomenon
Source: BMC Psychiatry. 2023 Dec 5;23:912. doi: 10.1186/s12888-023-05424-1 (PMC10698954; doi:10.1186/s12888-023-05424-1)
Supplement: Supplementary file 1 — Additional file 1: Table S1. The PID-5 trait statistics (n = 1264). Table S2. The PDQ-4 statistics (n = 1264). Table S3. The TEMPS-A statistics (n = 1264). Table S4. The TCI statistics (n = 1264). Table S5. The AFECTS statistics (n = 1264). Table S6. The PANAS statistics (n = 1264). Table S7. The statistics of somatization measures (n = 1264). Table S8. Demographic information and medical, psychiatric, and behavioral history of the groups. Table S9. Correlations between all personality systems and somatization factor. Table S10. Mean and standard deviation of personality traits between the groups. [file 12888_2023_5424_MOESM1_ESM.doc]

**Table S1.** The PID-5 trait statistics (n = 1264)

| Maladaptive traits | *N* item | *α* | Mean | SD | Skewness | Kurtosis |
| --- | --- | --- | --- | --- | --- | --- |
| Emotional liability | 7 | .76 | 1.16 | .62 | .13 | − .48 |
| Anxiousness | 9 | .85 | 1.14 | .66 | .44 | − .33 |
| Separation insecurity | 7 | .76 | .94 | .62 | .33 | − .57 |
| Withdrawal | 10 | .83 | .92 | .57 | .21 | − .46 |
| Anhedonia | 8 | .77 | 1.04 | .58 | .29 | − .53 |
| Intimacy avoidance | 6 | .68 | .98 | .60 | .39 | − .20 |
| Manipulativeness | 5 | .61 | .84 | .55 | .52 | .06 |
| Deceitfulness | 10 | .82 | .77 | .56 | .69 | − .02 |
| Grandiosity | 6 | .70 | 1.10 | .58 | .12 | − .50 |
| Irresponsibility | 7 | .72 | .76 | .54 | .62 | − .22 |
| Impulsivity | 6 | .82 | .87 | .67 | .61 | − .21 |
| Distractibility | 9 | .83 | .95 | .60 | .30 | − .36 |
| Unusual beliefs | 8 | .77 | .79 | .57 | .46 | − .50 |
| Eccentricity | 13 | .91 | .77 | .63 | .57 | −.42 |
| Perceptual dysregulation | 12 | .83 | .71 | .52 | .49 | − .41 |
| Attention seeking | 8 | .80 | 1.17 | .61 | .15 | − .36 |
| Callousness | 14 | .85 | .70 | .52 | .79 | .05 |
| Depressivity | 14 | .91 | .78 | .63 | .59 | − .44 |
| Hostility | 10 | .81 | 1.08 | .58 | .13 | − .45 |
| Perseveration | 9 | .79 | 1.04 | .56 | −.02 | − .62 |
| Restricted affectivity | 7 | .67 | .99 | .54 | .27 | − .46 |
| Rigid perfectionism | 10 | .75 | 1.27 | .54 | − .05 | − .03 |
| Risk-taking | 14 | .69 | 1.30 | .42 | .14 | .49 |
| Submissiveness | 4 | .56 | 1.10 | .60 | .13 | − .47 |
| Suspiciousness | 7 | .51 | 1.28 | .48 | .32 | .10 |

Abbreviation_ PID-5: Personality Inventory for DSM-5, SD: Standard deviation.

**Table S2.** The PDQ-4 statistics (n = 1264)

| Personality disorders | *N* item | *α* | Mean | SD | Skewness | Kurtosis |
| --- | --- | --- | --- | --- | --- | --- |
| Paranoid | 7 | .62 | 2.96 | 1.89 | .17 | −.78 |
| Schizoid | 7 | .49 | 2.15 | 1.56 | .52 | −.25 |
| Schizotypal | 9 | .60 | 2.47 | 1.88 | .71 | .01 |
| Antisocial | 8 | .65 | 1.53 | 1.68 | 1.12 | .74 |
| Borderline | 9 | .67 | 2.54 | 2.10 | .65 | −.37 |
| Narcissistic | 9 | .58 | 2.66 | 1.90 | .52 | −.23 |
| Histrionic | 8 | .55 | 2.60 | 1.78 | .47 | −.41 |
| Avoidant | 7 | .64 | 2.02 | 1.72 | .81 | .04 |
| Dependent | 8 | .70 | 1.84 | 1.88 | .96 | .25 |
| Obsessive-compulsive | 8 | .51 | 2.85 | 1.73 | .28 | −.36 |

Abbreviation_ Personality Diagnostic Questionnaire-4, SD: Standard deviation.

**Table S3.** The TEMPS-A statistics (n = 1264)

| Temperament traits | *N* item | *α* | Mean | SD | Skewness | Kurtosis |
| --- | --- | --- | --- | --- | --- | --- |
| TEMPS-A Depressive | 8 | .80 | 2.77 | 2.42 | .52 | − .90 |
| TEMPS-A Cyclothymic | 7 | .51 | 3.37 | 1.73 | .06 | − .64 |
| TEMPS-A Hyperthymic | 8 | .59 | 4.44 | 1.92 | −.14 | − .63 |
| TEMPS-A Irritable | 6 | .71 | 1.88 | 1.76 | .60 | −.75 |
| TEMPS-A Anxious | 6 | .62 | 1.40 | 1.44 | 1.02 | .40 |

Abbreviations_ TEMPS-A: Temperament Evaluation of Memphis, Pisa, Paris, and San Diego Autoquestionnaire, SD: Standard deviation.

**Table S4.** The TCI statistics (n = 1264)

| Temperament traits | *N* item | *α* | Mean | SD | Skewness | Kurtosis |
| --- | --- | --- | --- | --- | --- | --- |
| TCI Novelty seeking | 20 | .63 | 9.01 | 3.33 | .25 | −.22 |
| TCI Harm avoidance | 20 | .78 | 8.78 | 4.27 | .19 | −.46 |
| TCI Reward dependence | 15 | .43 | 8.28 | 2.30 | .04 | −.25 |
| TCI Persistence | 5 | .51 | 3.15 | 1.38 | − .54 | −.47 |

Abbreviations_ TCI: Temperament and Character Inventory, SD: Standard deviation.

**Table S5.** The AFECTS statistics (n = 1264)

| Temperament traits | *N* item | *α* | Mean | SD | Skewness | Kurtosis |
| --- | --- | --- | --- | --- | --- | --- |
| AFECTS Volition | 8 | .91 | 36.76 | 10.94 | −.29 | − .28 |
| AFECTS Anger | 8 | .83 | 28.49 | 9.37 | .10 | − .26 |
| AFECTS Inhibition | 8 | .62 | 32.83 | 7.04 | −.30 | .67 |
| AFECTS Sensitivity | 8 | .59 | 29.13 | 5.95 | −.18 | 1.05 |
| AFECTS Coping | 8 | .89 | 37.03 | 10.30 | −.32 | −.10 |
| AFECTS Control | 8 | .59 | 34.48 | 6.91 | −.39 | .87 |
| AFECTS Depressive | 1 | - | 1.99 | 1.19 | 1.01 | .10 |
| AFECTS Anxious | 1 | - | 2.39 | 1.21 | .43 | −.80 |
| AFECTS Apathetic | 1 | - | 1.99 | 1.09 | .87 | − .11 |
| AFECTS Cyclothymic | 1 | - | 2.63 | 1.21 | .21 | − .84 |
| AFECTS Dysphoric | 1 | - | 2.62 | 1.09 | .09 | − .60 |
| AFECTS Volatile | 1 | - | 2.26 | 1.14 | .62 | − .42 |
| AFECTS Obsessive | 1 | - | 3.09 | 1.06 | − .19 | − .44 |
| AFECTS Euthymic | 1 | - | 3.24 | 1.07 | −.30 | −.50 |
| AFECTS Hyperthymic | 1 | - | 3.19 | 1.13 | −.17 | − .61 |
| AFECTS Irritable | 1 | - | 2.53 | 1.09 | .29 | − .54 |
| AFECTS Disinhibited | 1 | - | 2.35 | 1.07 | .45 | − .43 |
| AFECTS Euphoric | 1 | - | 2.32 | 1.11 | .54 | − .44 |

Abbreviations_ AFECTS: Affective and Emotional Composite Temperament Scale, SD: Standard deviation.

**Table S6.** The PANAS statistics (n = 1264)

| Temperament traits | *N* item | *α* | Mean | SD | Skewness | Kurtosis |
| --- | --- | --- | --- | --- | --- | --- |
| Positive affect | 10 | .85 | 32.06 | 6.87 | −.02 | −.23 |
| Negative affect | 10 | .85 | 25.48 | 7.09 | .36 | −.12 |

Abbreviations_ PANAS: Positive and Negative Affect Schedule, SD: Standard deviation.

**Table S7.** The statistics of somatization measures (n = 1264)

| Somatization scale | *N* item | *α* | Mean | SD | Skewness | Kurtosis |
| --- | --- | --- | --- | --- | --- | --- |
| SOMS-7 | 47 | .94 | 17.93 | 16.39 | 1.43 | 1.93 |
| SCL-90-R | 12 | .90 | 13.12 | 9.91 | .78 | .01 |
| PHQ-15 | 15 | .84 | 6.72 | 5.21 | 1.05 | 1.30 |
| SHAI | 18 | .84 | 17.08 | 8.28 | .63 | .23 |

Abbreviation_ SOMS-7: Screening for Somatic Symptom Disorders-7, PHQ-15: Patient Health Questionnaire-15, SCL-90-R: Symptom Checklist-90-Revised Form, SHAI: Short Health Anxiety Inventory, SD: Standard deviation.

**Table S8.** Demographic information and medical, psychiatric, and behavioral history of the groups

| Variables | Total (N=1264) | SSRD (n=257) | Non-SSRD (n=1007) | Statics | *P* |
| --- | --- | --- | --- | --- | --- |
| Sex, female (%)^a^ | 830 (65.7) | 182 (70.8) | 648 (64.3) | 3.799 | .051 |
| Education (%)^a^ |  |  |  | 61.287 | <.001 |
| Under diploma | 159 (12.5) | 66 (25.7) | 93 (9.2) |  |  |
| Diploma | 354 (28.1) | 64 (24.9) | 290 (28.8) |  |  |
| Academic | 751 (59.4) | 127 (49.4) | 624 (62) |  |  |
| Job (%)^a^ |  |  |  | 39.688 | <.001 |
| College student | 289 (22.9) | 72 (28) | 217 (21.5) |  |  |
| Employed | 220 (17.4) | 26 (10.1) | 194 (19.3) |  |  |
| Self-employed | 209 (16.5) | 28 (10.9) | 181 (18) |  |  |
| Housekeeper | 334 (26.4) | 97 (37.8) | 237 (23.5) |  |  |
| Retired | 38 (3.1) | 5 (1.9) | 33 (3.3) |  |  |
| Other | 174 (13.7) | 29 (11.3) | 145 (14.4) |  |  |
| Marital status (%)^a^ |  |  |  | 7.022 | .135 |
| Single | 557 (44.1) | 108 (42) | 449 (44.6) |  |  |
| Married | 637 (50.4) | 129 (50.2) | 508 (50.4) |  |  |
| Widow/divorced | 70 (5.5) | 20 (7.8) | 50 (5) |  |  |
| Medical history (%)^a^ |  |  |  |  |  |
| Diabetes | 40 (3.2) | 15 (5.8) | 25 (2.5) | 3.857 | .050 |
| Hypertension | 58 (4.6) | 17 (6.6) | 41 (4.1) | 1.286 | .257 |
| Cardiovascular | 29 (2.3) | 8 (3.1) | 21 (2.1) | .692 | .405 |
| Epilepsy | 14 (1.1) | 2 (.8) | 12 (1.2) | .200 | .655 |
| Migraine | 97 (7.7) | 20 (7.8) | 7 (7.6) | .026 | .873 |
| Kidney disease | 14 (1.1) | 5 (1.9) | 9 (.9) | 1.286 | .257 |
| Gastrointestinal disease | 53 (4.2) | 11 (4.3) | 42 (4.2) | .001 | .999 |
| Sinusitis | 101 (8) | 18 (7) | 83 (8.2) | .231 | .631 |
| Other | 311 (24.6) | 65 (25.3) | 246 (24.4) | .031 | .860 |
| Psychiatric history (%)^a^ |  |  |  |  |  |
| Anxiety disorders | 191 (15.1) | 90 (35) | 101 (10) | 35.310 | <.001 |
| Affective disorders | 192 (15.2) | 74 (28.8) | 118 (11.7) | 18.615 | <.001 |
| Personality disorders | 401 (31.7) | 131 (51) | 270 (26.8) | 19.220 | <.001 |
| Childhood trauma | 53 (4.2) | 13 (5.1) | 40 (4) | .391 | .532 |
| Psychiatric treatment (%)^a^ |  |  |  | 5.890 | .117 |
| 1-month | 63 (5) | 17 (6.6) | 46 (4.6) |  |  |
| 12-month | 43 (3.4) | 12 (4.7) | 31 (3.1) |  |  |
| Lifetime | 58 (4.6) | 16 (6.2) | 42 (4.2) |  |  |
| Behavioral history (%)^a^ |  |  |  |  |  |
| Smoking | 171 (14.3) | 41 (16) | 140 (13.9) | .779 | .677 |
| Alcohol use | 128 (10.1) | 22 (8.6) | 106 (6) | .921 | .631 |
| Substance use | 23 (1.8) | 5 (2) | 18 (1.8) | 3.982 | .137 |
| Age (Mean ± SD)^b^ | 33.73 ± 11.29 | 34.33 ± 11.85 | 33.57 ± 11.14 | -.964 | .335 |

Note: Significance of differences was assessed using (a) chi-square test and (b) t-test; (c) except somatization.

Abbreviations_ SD: standard deviation, SSRD: Somatic Symptom and Related Disorders.

**Table S9.** Correlations between all personality systems and somatization factor

| Personality systems | r | P |
| --- | --- | --- |
| DSM-5 PD categories |  |  |
| Paranoid | .201 | < .001 |
| Schizoid | .198 | < .001 |
| Schizotypal | .245 | < .001 |
| Antisocial | .196 | < .001 |
| Borderline | .341 | < .001 |
| Narcissistic | .132 | < .001 |
| Histrionic | .181 | < .001 |
| Avoidant | .245 | < .001 |
| Dependent | .297 | < .001 |
| Obsessive-compulsive | .200 | < .001 |
| DSM-5 PD composites |  |  |
| Schizotypal (6 facets) | .407 | < .001 |
| Antisocial (7 facets) | .317 | < .001 |
| Borderline (7 facets) | .480 | < .001 |
| Narcissistic (2 facets) | .146 | < .001 |
| Avoidant (4 facets) | .462 | < .001 |
| Obsessive-compulsive (4 facets) | .353 | < .001 |
| DSM-5 trait model |  |  |
| Negative affectivity (3 facets) | .459 | < .001 |
| Detachment (3 facets) | .395 | < .001 |
| Antagonism (3 facets) | .191 | < .001 |
| Disinhibition (3 facets) | .404 | < .001 |
| Psychoticism (3 facets) | .396 | < .001 |
| ICD-11 trait model |  |  |
| Negative affectivity (4 facets) | .506 | < .001 |
| Detachment (3 facets) | .309 | < .001 |
| Dissociality (5 facets) | .246 | < .001 |
| Disinhibition (4 facets) | .384 | < .001 |
| Anankastia (2 facets) | .352 | < .001 |
| TEMPS |  |  |
| Depressive | .551 | < .001 |
| Cyclothymic | .412 | < .001 |
| Hyperthymic | − .125 | < .001 |
| Irritable | .554 | < .001 |
| Anxious | .433 | < .001 |
| TCI |  |  |
| Novelty seeking | .140 | < .001 |
| Harm avoidance | .332 | < .001 |
| Reward dependence | .007 | .808 |
| Persistence | .038 | .181 |
| Self-directedness | − .412 | < .001 |
| Cooperativeness | − .243 | < .001 |
| Self-transcendence | .055 | .050 |
| AFECT |  |  |
| Volition | − .339 | < .001 |
| Anger | .161 | < .001 |
| Inhibition | .050 | .077 |
| Sensitivity | − .031 | .266 |
| Coping | − .269 | < .001 |
| Control | − .105 | < .001 |
| Depressive | .432 | < .001 |
| Anxious | .431 | < .001 |
| Apathetic | .350 | < .001 |
| Cyclothymic | .363 | < .001 |
| Dysphoric | .424 | < .001 |
| Volatile | .408 | < .001 |
| Obsessive | .021 | .455 |
| Euthymic | − .202 | < .001 |
| Hyperthymic | − .213 | < .001 |
| Irritable | .180 | < .001 |
| Disinhibited | .270 | < .001 |
| Euphoric | .178 | < .001 |
| PANAS |  |  |
| Positive affect | − .247 | < .001 |
| Negative affect | .489 | < .001 |

**Abbreviations_** AFECTS: Affective and Emotional Composite Temperament Scale, DSM: Diagnostic and Statistical Manual of Mental Disorders, ICD: International Classification of Diseases, PANAS: Positive and Negative Affect Schedule, PID-5: Personality Inventory for DSM-5, TCI: Temperament and Character Inventory, TEMPS: Temperament Evaluation of Memphis, Pisa, Paris, and San Diego Autoquestionnaire, SHAI: Short Health Anxiety Inventory, SOMS-7: Screening for Somatic Symptom Disorders, SCL-90-R: Revised Form of Symptom Checklist-90, PDQ-4: Fourth Edition of the Personality Diagnostic Questionnaire.

**Table S10.** Mean and standard deviation of personality traits between the groups

| Personality traits  (Mean ± SD) | Total (N=1264) | SSRD (n=257) | Non-SSRD (n=1007) | *t*-test | *P* |
| --- | --- | --- | --- | --- | --- |
| DSM-5 PD categories |  |  |  |  |  |
| Paranoid | 2.96 ± 1.89 | 3.47 ± 1.88 | 2.83 ± 1.87 | − 4.865 | < .001 |
| Schizoid | 2.15 ± 1.56 | 2.71 ± 1.58 | 2.01 ± 1.52 | − 6.385 | < .001 |
| Schizotypal | 2.47 ± 1.88 | 3.35 ± 1.94 | 2.24 ± 1.79 | − 8.289 | < .001 |
| Antisocial | 1.53 ± 1.68 | 2.24 ± 1.78 | 1.35 ± 1.60 | − 7.311 | < .001 |
| Borderline | 2.54 ± 2.10 | 3.64 ± 2.16 | 2.26 ± 1.99 | − 9.315 | < .001 |
| Narcissistic | 2.66 ± 1.90 | 3.16 ± 1.91 | 2.53 ± 1.88 | − 4.730 | < .001 |
| Histrionic | 2.60 ± 1.78 | 3.13 ± 1.79 | 2.47 ± 1.75 | − 5.278 | < .001 |
| Avoidant | 2.02 ± 1.72 | 2.76 ± 1.80 | 1.83 ± 1.65 | − 7.535 | < .001 |
| Dependent | 1.84 ± 1.88 | 2.82 ± 1.93 | 1.59 ± 1.78 | − 9.229 | < .001 |
| Obsessive-compulsive | 2.85 ± 1.73 | 3.37 ± 1.79 | 2.70 ± 1.69 | − 5.226 | < .001 |
| DSM-5 PD composites |  |  |  |  |  |
| Schizotypal (6 facets) | .91 ± .44 | 1.18 ± .40 | .84 ± .42 | − 12.145 | < .001 |
| Antisocial (7 facets) | .90 ± .42 | 1.13 ± .40 | .84 ± .41 | − 9.963 | < .001 |
| Borderline (7 facets) | 1.04 ± .44 | 1.34 ± .37 | .96 ± .42 | − 14.272 | < .001 |
| Narcissistic (2 facets) | 1.14 ± .53 | 1.26 ± .51 | 1.11 ± .53 | − 4.220 | < .001 |
| Avoidant (4 facets) | 1.02 ± .47 | 1.33 ± .39 | .94 ± .45 | − 13.760 | < .001 |
| Obsessive-compulsive (4 facets) | 1.07 ± .43 | 1.29 ± .35 | 1.01 ± .43 | − 10.784 | < .001 |
| DSM-5 trait model |  |  |  |  |  |
| Negative affectivity (3 facets) | 1.08 ± .53 | 1.43 ± .45 | .99 ± .50 | − 13.868 | < .001 |
| Detachment (3 facets) | .98 ± .47 | 1.26 ± .41 | .91 ± .46 | − 11.563 | < .001 |
| Antagonism (3 facets) | .90 ± .47 | 1.06 ± .49 | .86 ± .46 | − 6.006 | < .001 |
| Disinhibition (3 facets) | .86 ± .51 | 1.15 ± .45 | .79 ± .50 | − 11.138 | < .001 |
| Psychoticism (3 facets) | .76 ± .52 | 1.07 ± .48 | .68 ± .49 | − 11.689 | < .001 |
| ICD-11 trait model |  |  |  |  |  |
| Negative affectivity (4 facets) | 1.09 ± .49 | 1.43 ± .42 | 1.00 ± .47 | − 14.212 | < .001 |
| Detachment (3 facets) | .97 ± .48 | 1.20 ± .43 | .91 ± .47 | − 9.473 | < .001 |
| Dissociality (5 facets) | .98 ± .44 | 1.17 ± .42 | .93 ± .43 | − 8.056 | < .001 |
| Disinhibition (4 facets) | .97 ± .43 | 1.20 ± .40 | .91 ± .42 | − 10.071 | < .001 |
| Anankastia (2 facets) | 1.15 ± .49 | 1.37 ± .41 | 1.10 ± .50 | − 9.089 | < .001 |
| TEMPS-A |  |  |  |  |  |
| Depressive | 2.77 ± 2.42 | 4.46 ± 2.40 | 2.33 ± 2.22 | − 13.472 | < .001 |
| Cyclothymic | 3.37 ± 1.73 | 4.06 ± 1.67 | 3.19 ± 1.70 | − 7.373 | < .001 |
| Hyperthymic | 4.44 ± 1.92 | 4.12 ± 1.77 | 4.53 ± 1.95 | 3.032 | .002 |
| Irritable | 1.88 ± 1.76 | 3.21 ± 1.59 | 1.54 ± 1.63 | − 14.708 | < .001 |
| Anxious | 1.40 ± 1.44 | 2.33 ± 1.38 | 1.16 ± 1.36 | − 12.317 | < .001 |
| TCI |  |  |  |  |  |
| Novelty seeking | 9.01 ± 3.33 | 9.40 ± 3.27 | 8.91 ± 3.34 | − 2.096 | .036 |
| Harm avoidance | 8.78 ± 4.27 | 10.93 ± 3.82 | 8.23 ± 4.41 | − 9.322 | < .001 |
| Reward dependence | 8.28 ± 2.30 | 8.33 ± 2.18 | 8.27 ± 2.34 | − .348 | .728 |
| Persistence | 3.15 ± 1.38 | 3.23 ± 1.21 | 3.13 ± 1.41 | − .987 | .324 |
| Self-directedness | 13.23 ± 5.08 | 10.67 ± 4.34 | 13.89 ± 5.06 | 10.231 | < .001 |
| Cooperativeness | 16.66 ± 4.16 | 14.43 ± 4.20 | 17.23 ± 3.96 | 9.643 | < .001 |
| Self-transcendence | 9.37 ± 2.68 | 9.38 ± 2.47 | 9.37 ± 2.73 | − .37 | .971 |
| AFECTS |  |  |  |  |  |
| Volition | 36.76 ± 10.94 | 30.76 ± 10.04 | 38.29 ± 10.63 | 10.238 | < .001 |
| Anger | 28.49 ± 9.37 | 31.13 ± 9.02 | 27.81 ± 9.35 | − 5.109 | < .001 |
| Inhibition | 32.83 ± 7.04 | 33.30 ± 7.12 | 32.71 ± 7.02 | − 1.185 | .236 |
| Sensitivity | 29.13 ± 5.95 | 28.94 ± 6.31 | 29.18 ± 7.02 | .581 | .562 |
| Coping | 37.03 ± 10.30 | 32.90 ± 8.70 | 38.09 ± 10.41 | 7.356 | < .001 |
| Control | 34.48 ± 6.91 | 33.72 ± 6.80 | 34.68 ± 6.92 | 1.976 | .048 |
| Depressive | 1.99 ± 1.19 | 2.69 ± 1.40 | 1.80 ± 1.06 | − 11.054 | < .001 |
| Anxious | 2.39 ± 1.21 | 3.16 ± 1.18 | 2.20 ± 1.14 | − 11.954 | < .001 |
| Apathetic | 1.99 ± 1.09 | 2.57 ± 1.22 | 1.84 ± 1.00 | − 9.892 | < .001 |
| Cyclothymic | 2.63 ± 1.21 | 3.13 ± 1.13 | 2.50 ± 1.20 | − 7.691 | < .001 |
| Dysphoric | 2.62 ± 1.09 | 3.11 ± 1.01 | 2.50 ± 1.07 | − 8.347 | < .001 |
| Volatile | 2.26 ± 1.14 | 2.89 ± 1.19 | 2.10 ± 1.08 | − 10.272 | < .001 |
| Obsessive | 3.09 ± 1.06 | 3.09 ± 1.14 | 3.09 ± 1.04 | .104 | .917 |
| Euthymic | 3.24 ± 1.07 | 2.87 ± 1.05 | 3.34 ± 1.06 | 6.250 | < .001 |
| Hyperthymic | 3.19 ± 1.13 | 2.79 ± 1.15 | 3.30 ± 1.11 | 6.516 | < .001 |
| Irritable | 2.53 ± 1.09 | 2.86 ± 1.13 | 2.45 ± 1.06 | − 5.360 | < .001 |
| Disinhibited | 2.35 ± 1.07 | 2.79 ± 1.07 | 2.24 ± 1.04 | − 7.625 | < .001 |
| Euphoric | 2.32 ± 1.11 | 2.63 ± 1.12 | 2.25 ± 1.09 | − 4.932 | < .001 |
| PANAS |  |  |  |  |  |
| Positive affect | 32.06 ± 6.87 | 28.95 ± 6.84 | 32.85 ± 6.87 | 9.138 | < .001 |
| Negative affect | 25.48 ± 7.09 | 29.41 ± 6.84 | 24.47 ± 6.80 | − 10.355 | < .001 |
| Somatization scales |  |  |  |  |  |
| SOMS-7 somatization | 17.93 ± 16.39 | 34.16 ± 21.31 | 13.79 ± 11.73 | − 20.522 | < .001 |
| SCL90-somatization | 13.12 ± 9.91 | 21.14 ± 10.04 | 11.07 ± 8.78 | − 15.916 | < .001 |
| PHQ-15 somatization | 6.72 ± 5.21 | 10.52 ± 5.80 | 5.75 ± 4.57 | − 12.235 | < .001 |
| SHAI health anxiety | 17.08 ± 8.28 | 27.65 ± 6.67 | 14.38 ± 6.24 | − 29.985 | < .001 |

Note: The significance of differences was assessed using a t-test.

**Abbreviations_** AFECTS: Affective and Emotional Composite Temperament Scale, DSM: Diagnostic and Statistical Manual of Mental Disorders, ICD: International Classification of Diseases, PANAS: Positive and Negative Affect Schedule, PID-5: Personality Inventory for DSM-5, TCI: Temperament and Character Inventory, TEMPS: Temperament Evaluation of Memphis, Pisa, Paris, and San Diego Autoquestionnaire, SSRD: somatic symptom and related disorders.
